# Comparative analysis of sex-dependent serum metabolomic patterns across the lifespan of rhesus macaques

**DOI:** 10.3389/fgene.2025.1655325

**Published:** 2025-08-25

**Authors:** Ludwig A. P. Metzler, Robinson W. Goy, Jeanette M. Metzger, Marina E. Emborg, Amita Kapoor

**Affiliations:** ^1^ Wisconsin National Primate Research Center, University of Wisconsin, Madison, WI, United States; ^2^ Department of Medical Physics, University of Wisconsin, Madison, WI, United States

**Keywords:** metabolomics, age, rhesus macaques, lipids, amino acids, sex differences

## Abstract

**Introduction:**

Aging is accompanied by systemic metabolic changes that contribute to disease susceptibility and functional decline. Sex differences in aging have been reported in humans, yet their mechanistic basis remains poorly understood. Due to their physiological similarity to humans, rhesus macaques are a powerful translational model to investigate sex-specific metabolomic aging under controlled conditions.

**Methods:**

Targeted serum metabolomics were conducted in 58 rhesus (35 females, 23 males), ranging from 1.66 to 25.71 years of age, quantifying 513 metabolites spanning lipids, amino acids, and related compounds. Multivariate, univariate, and generalized additive model (GAM) analyses were performed to evaluate age-associated trajectories and test for sex differences.

**Results:**

Age-related changes in both sexes were identified in metabolites related to hormones (e.g., DHEAS), amino acid biosynthesis and catabolism (e.g., beta-alanine, sarcosine, t4-OH-pro), and energy metabolism (e.g., hexose). Sex affected age-related metabolic trajectories in lipids, amino acids and related compounds, and gut microbial species. Females demonstrated a profound increase in serum triglycerides (TGs), amino acids, and other small molecules, while males exhibited a heterogenous profile with changes in lipids, but no TGs were affected. Males also exhibited altered levels of amino acids and related metabolites, hormones, gut microbial metabolites, and energy-associated metabolites.

**Conclusion:**

These results highlight pronounced sex differences in metabolomic aging trajectories in rhesus macaques, particularly in lipid and amino acid metabolism. These findings underscore the importance of incorporating sex as a biological variable in aging studies and support the utility of rhesus macaques for identifying conserved, sex-specific biomarkers of biological aging.

## 1 Introduction

Aging is characterized by a progressive decline in physiological function, accompanied by alterations in cellular signaling, metabolism, and the loss of homeostatic regulation, all of which increase the risk of morbidity and mortality, including neurodegenerative disease. As the global population of adults aged 65 and older continues to grow, there is a corresponding rise in chronic conditions associated with aging (United Nations Department of Economic and Social Affairs, 2023). Sex differences are well documented in human aging; women tend to live longer than men but experience a greater lifetime burden of chronic diseases, including osteoporosis, dementia, and other age-related neurodegenerative disorders (DuMont et al., 2023; Patwardhan et al., 2024). Although some of this elevated disease burden can be attributed to women’s greater longevity, emerging evidence suggests that biological factors such as genetics and hormone regulation also contribute to sex-specific aging trajectories (Bronikowski et al., 2022).

Metabolomics, the comprehensive study of metabolite profiles in biological samples, is emerging as a powerful tool for investigating biochemical changes associated with aging (Clish, 2015). The metabolome reflects the functional endpoints of a complex network of biological processes, shaped by genomic, epigenomic, transcriptomic, proteomic, and environmental influences. Early identification of age-related metabolic alterations has the potential to reveal targets for preserving physiological function and delaying the onset of age-associated diseases. Aging is accompanied by disruptions in energy balance, redox homeostasis, and inflammatory regulation and it has been suggested that younger individuals maintain more flexible and adaptive metabolic networks that support health and resilience (Gilbert, 2000; Jasbi et al., 2023). Human metabolomic studies have demonstrated age-related shifts in lipid, amino acid, and other small-molecule metabolism (Darst et al., 2019). Importantly, when sex is considered as a factor, males and females exhibited distinct patterns of metabolomic aging (e.g., Menni et al., 2013; Darst et al., 2019), underscoring the utility of metabolomics for identifying pathways that contribute to sex-specific trajectories of aging.

Rhesus macaques are widely recognized as valuable models for human aging research due to their close phylogenetic relationship with humans and similar patterns of age-related physiological decline (Simmons, 2016; Colman, 2018; Metzler et al., 2025). Like humans, female rhesus macaques exhibit greater longevity than males (Huber et al., 2024). Critically, captive rhesus macaques are housed under controlled conditions with standardized diets and environmental enrichment, reducing many of the lifestyle and environmental confounders that limit mechanistic insights in human studies. For example, diet and its influence on the gut microbiome can account for up to 50% of the explained variance in human metabolomic data (Bar et al., 2020). Yet, metabolomic studies in non-human primates are still rare. Hoffman et al. (2019) examined sex- and age-related changes in the serum metabolome of common marmoset monkeys and identified differences mainly associated with age. It should be noted that marmosets are a New World monkey species, further phylogenetically divergent from humans compared to macaques. To our knowledge, the study by Chen et al. (2023) is currently the only available peer-reviewed report on serum-based metabolomics in aging rhesus. The investigators applied untargeted metabolomics to identify age-related changes in amino acids and lipids only in male monkeys.

Here, we report the characterization of age-related changes in the serum metabolome of rhesus macaques using a cross-sectional design, with a specific focus on identifying metabolites that differ by sex across age. The goal of the study was to uncover sex-specific metabolic signatures that may underlie divergent aging trajectories in male and female macaques and generate a foundation to inform translational research and validate the use of this species as a preclinical model of aging and disease.

## 2 Methods

### 2.1 Subjects

All procedures were performed in accordance with the NIH guide for the Care and Use of Laboratory Animals and under the approval of the Vice Chancellor Office for Research and Graduate Education Institutional Animal Care and Use Committee (Protocol G005091). Animals were housed in enclosures that meet the requirements specified in the Animal Welfare Act Regulations and the Guide for the Care and Use of Laboratory Animals Guide. All animals were evaluated by veterinary staff or trained animal care staff at least twice daily for signs of pain, distress, and illness.

The rhesus macaques used in this study were cross-sectional samples of convenience from the colony at the Wisconsin National Primate Research Center (WNPRC). The study included 58 rhesus macaques, 35 females and 23 males, ranging in age from 1.66 to 25.71. Age, weight, and parity (females only) information for individual macaques in the study are presented in Supplementary Table S1.

**FIGURE 1:**
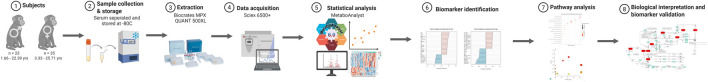
Workflow schematic of the study. Steps are shown as sequential panels and arrows indicate the process of flow between panels.

Rhesus macaques were sedated for blood draws with ketamine (10 mg/kg i.m.) and their vital signs were monitored until they recovered from anesthesia. Blood was collected in serum separator tubes, allowed to clot for 30 min, and centrifuged. All serum samples were stored at −80 °C until assay. A targeted metabolomics approach was applied to the serum samples for quantification of a broad panel of biologically relevant small molecules and lipids, including amino acids, biogenic amines, and lipid subclasses. A workflow of the study is presented in Figure 1.

### 2.2 Metabolomic extraction and analysis

MxP Quant 500 and Quant 500 XL metabolomic kits were obtained from Biocrates (Innsbruck, Austria). Rhesus macaque serum was prepared according to the kit instructions supplied from the manufacturer. Briefly, 10 µL of serum, calibrators, and quality control samples were loaded onto the Quant 500 and Quant 500 XL plates. Samples were allowed to dry under a constant stream of nitrogen and derivatized using phenyl isothiocyanate and eluted from the filter using 5 mM ammonium acetate in methanol. Three plates of extracts were generated-one for liquid chromatography tandem mass spectrometry (LC-MS/MS) and two for flow injection analysis (FIA). Extracts were run on a 6,500+ triple quadrupole mass spectrometer (SCIEX, Framingham, MA) equipped with an ExionLC liquid chromatography system. For methods using liquid chromatography, resolution was achieved using a supplier-provided analytical column. Data processing was done using Biocrates WebIDQ software. Quality of data was ensured using the supplier-provided quality control material, which encompassed coefficient of variation (CV), peak retention time, and internal standard area counts.

### 2.3 Statistical analysis

Metabolomics data were pre-processed following established protocols for metabolomic analysis (Chen et al., 2022). All samples were processed and analyzed in a single run to eliminate the need for batch correction. After metabolite concentrations were obtained, two exclusion criteria were applied: (1) only metabolites detected in at least 80% of all samples were retained and (2) metabolites with a coefficient of variation (CV) greater than 20% across replicates were excluded to minimize the influence of technical variability (Floegel et al., 2011).

The resulting dataset was log-transformed and mean-centered to reduce variance and approximate a normal distribution. All data processing and statistical analyses were conducted using MetaboAnalyst 5.0 (Pang et al., 2021). The Metadata Analysis module was used to examine associations between metabolite levels and group-defining variables. Each sample was linked to an animal-specific ID, which was further associated with relevant metadata, including sex (categorical), age (continuous), and weight (continuous).

Principal component analysis (PCA) was conducted to explore multivariate structure and identify dominant patterns of variance using the Metadata Analysis module in MetaboAnalyst 5.0 (Pang et al., 2021). The first three principal components (PC1 – PC3) were retained, and their corresponding score values were exported for further analysis. Associations between component scores and age were assessed using Pearson correlations (Price et al., 2011) in GraphPad Prism 10. Analyses were stratified by sex to examine sex-specific relationships between age and multivariate metabolic profiles.

To identify individual metabolites associated with age, Pearson correlation coefficients (r) were calculated between each metabolite and chronological age. This analysis was used to assess linear relationships and rank metabolites by strength of association. The top 25 metabolites, based on absolute correlation values, were retained for further interpretation. Statistical significance was determined using false discovery rate (FDR) correction with the Benjamini–Hochberg procedure, applying a significance threshold of FDR < 0.05.

Heatmaps were generated using MetaboAnalyst utilizing z-score normalized values while controlling for weight. Hierarchical clustering was applied to both samples and metabolites using Euclidean distance and complete linkage. Metadata for age and weight were overlaid as color-coded annotations above each heatmap to aid in the interpretation of covariate-related patterns. For visualization, the top 80 metabolites from females and the top 50 from males were selected based on their contribution to age-related clustering.

Finally, to characterize and visualize age-related changes in serum metabolite concentrations, generalized additive models (GAMs) with thin plate regression splines were used for selected metabolites that were significant in the correlation model for both sexes. Prior to the implementation of the GAMs, raw data was winsorized to minimize outlier effect on the model. Outlier detection was performed through Tukey’s Fences. All models were fitted using the maximum likelihood (ML) method. For each metabolite, the following model structure was applied:

Metabolite∼ s(Age,by=sex)+sex (1)

To assess whether males and females exhibited significantly different age trajectories for a given metabolite, model 1 was compared to a nested version of the model without sex-specific smooths:

Metabolite∼ s(Age)+sex (2)

Model comparisons were performed using F-based ANOVA. All modelling and visualization were conducted in R using the mgcv package (v4.4.1), with fitted values and 95% confidence intervals plotted separately for males and females. A significance level of p < 0.05 was considered as statistically significant.

## 3 Results

### 3.1 Unsupervised metabolite analysis

After application of exclusion criteria, 513 serum metabolites were quantified. To capture broad metabolic shifts, PCA was performed on all metabolites.

**FIGURE 2:**
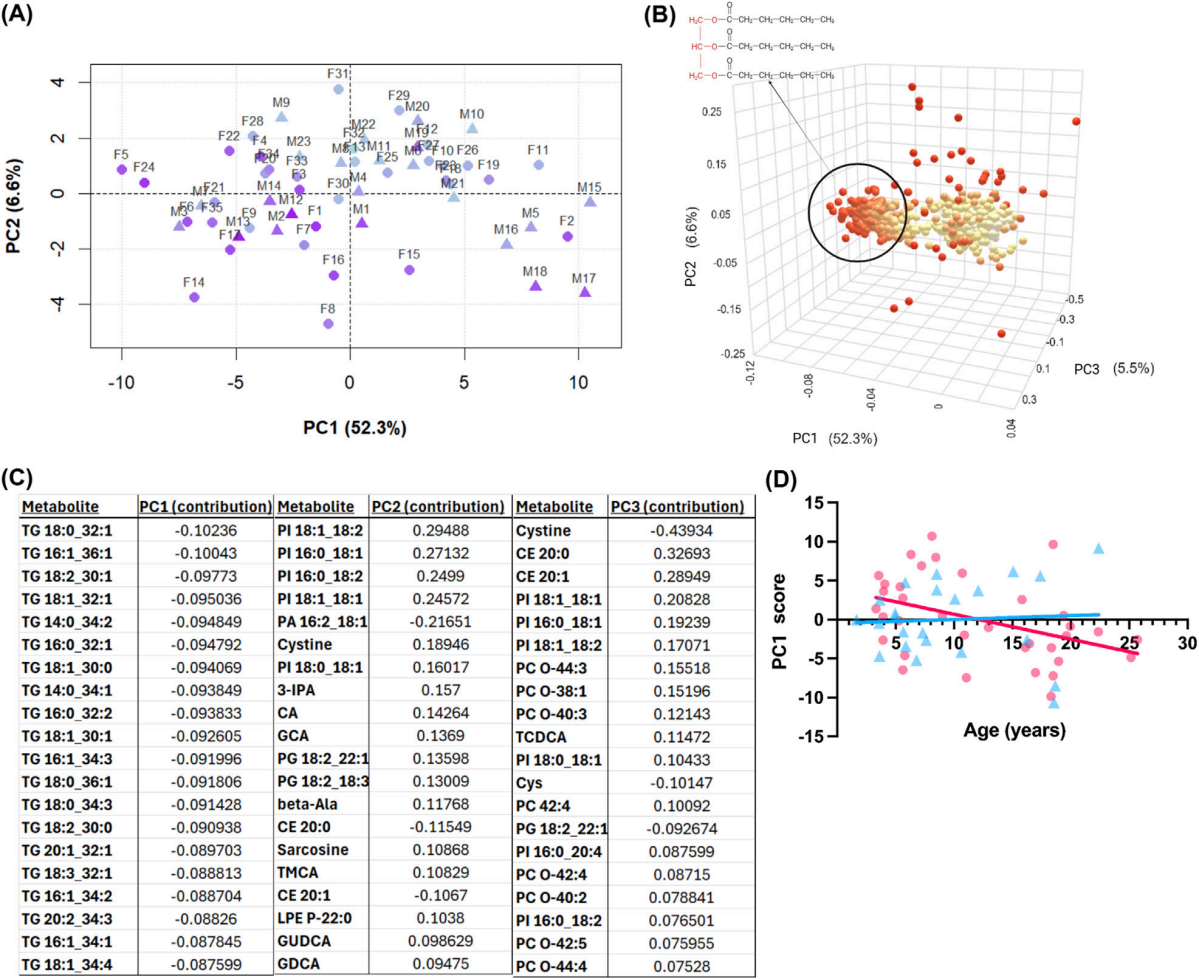
Principal component analysis (PCA) of serum metabolomics data from both sexes. **(A)** score plots of independent principal components (PCs) 1-2. Axis labels show percent variance explained by each PC. Pyramids, males; spheres, females. Age is visualized by a light-blue to purple color transition with light-blue indicating young age and purple showing old age. **(B)** Three-dimensional loadings plot showing each metabolite’s contribution to the PCs. Point color shows magnitude of contribution, with red indicating greater magnitude of contribution. The circled cluster highlights triglycerides impacting PC1; triglyceride general chemical structure. **(C)** Table of top metabolite loadings (by net contribution) for PC1, PC2, and PC3. **(D)** PC1 score versus chronological age (years), with females shown as pink circles and males as blue triangles.

The first five PCs accounted for a combined 72.9% of the total variance, with PC1 alone explaining 52.3%. In a pairwise plot of PC1 vs. PC2, there was a clear separation by age for PC1 (Figure 2A). A 3D scores plot (Figure 2B) was generated to visualize the metabolite contributions to the top three PCs. PC1 was almost exclusively driven by triglyceride (TG) species. PCs 2 and 3 explained smaller proportions of the variance (6.6% and 5.5%, respectively; Figure 2B). The top 20 metabolites contributing to PC1 are shown in Figure 2C and include primarily TGs, followed by other lipid classes, amino acids, amino acid-related metabolites, and small molecules. Figure 2C shows that PC1, which separates younger and older individuals, was heavily driven by TGs and the glycerophospholipid subclass phosphatidylcholines, while PC2 highlighted contributions from the glycerophospholipid subclass phosphatidylinositols. To determine if there was an effect of sex on the age-separated PC1, scores were stratified by sex and correlated with age (Figure 2D). In females, PC1 scores showed a significant negative correlation with age (r = −0.4208, p = 0.0118), whereas no association was observed in males (r = 0.0574, p = 0.7948). No other PCs were significantly correlated with age in either sex. These findings suggest that the primary axis of variance in the serum metabolome—driven by TGs—is selectively age-associated in females, despite the absence of overt sex-based clustering in the PCA.

### 3.2 Metabolite correlations with age by sex

**FIGURE 3:**
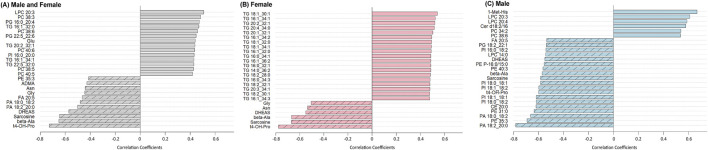
Correlation coefficients between serum metabolite levels and age, stratified by sex. For **(A)**, combined group (both sexes): metabolites with the strongest positive (dark gray bars) and negative (hashed gray bars) Pearson correlations with age. **(B)** Females only: metabolites positively correlated with age are shown in red bars, and those negatively correlated are hashed red bars. **(C)** Males only: metabolites positively correlated with age are shown in blue bars, and those negatively correlated are hashed blue bars. A Benjamini–Hochberg adjusted p FDR < 0.05 was considered significant.

Of the 513 analyzed metabolites, 87 exhibited a significant correlation with age, with 68 increasing and 19 decreasing (Supplementary Table S3). The 25 metabolites with the strongest age associations, independent of sex, are presented in Figure 3A. A variety of lipid species were positively correlated with age, including glycerolipid subclasses such as lysophosphatidylcholines, phosphatidylcholines, phosphatidylglycerols, and the glycerolipid subclass TGs. Age-related changes in proteinogenic amino acids were primarily characterized by declines observed in both sexes, including methionine (r = −0.3509, FDR p = 0.048), threonine (r = −0.3962, FDR p = 0.032), tyrosine (r = −0.4102, FDR p = 0.027), glycine (r = −0.4434, FDR p = 0.018), and asparagine (r = −0.4412, FDR p = 0.018). In contrast, glutamate (r = 0.4398, FDR p = 0.018) and aspartate (r = 0.3784, FDR p = 0.035) increased with age, indicating that not all proteinogenic amino acids followed the same trajectory. Amino acid-related metabolites, including trans-4-hydroxyproline (t4-OH-pro; r = −0.7278, FDR p < 0.005), beta-alanine (r = −0.6508, FDR p < 0.005), and sarcosine (r = −0.6475, FDR p < 0.005) were negatively associated with age, along with the steroid hormone dehydroepiandrosterone sulfate (DHEAS; r = −0.5713, FDR p < 0.005), hexose (r = 0.3685, FDR p = 0.042), and 3-Indolepropionic acid (3-IPA; r = −0.3492, FDR p 0.048).

To identify metabolites associated with age in a sex-specific manner, correlation analyses were stratified by sex. In females, all 19 metabolites that showed positive associations with age were TGs, consistent with PCA results (Figure 3B). Several amino acids, related metabolites, and hormones that were negatively associated with age in the combined analysis also remained significant in females, including asparagine (r = −0.5363, FDR p = 0.0079), glycine (r = −0.5051, FDR p = 0.0079), beta-alanine (r = −0.6672, FDR p < 0.005), sarcosine (r = −0.6691, FDR p < 0.005), t4-OH-pro (r = −0.7772, FDR p < 0.005), and DHEAS (r = −0.5509, FDR p = 0.0060, Figure 3B).

In males (Figure 3C), the metabolites that remained significantly associated with age from the combined and female analyses were non-proteinogenic amino acids beta-alanine (=−0.5701, FDR p = 0.0075), t4-OH-Pro (r = −0.5983, FDR p < 0.005), sarcosine (r = −0.5845, FDR p < 0.005), and the hormone DHEAS (r = −0.5501, FDR p = 0.0093). Unique to males, the amino acid-related metabolite 1-methylhistidine (1-Met-His; r = 0.6693, FDR p < 0.005) had a strong positive correlation with age. Multiple lipid classes showed male-specific associations including choline-containing glycerophospholipids such as phosphatidylcholines and lysophosphatidylcholines, as well as the sphingolipid species ceramides. Additional lipid species negatively associated with age included phosphatidylinositols, phosphatidic acids, different species of lysophosphatidylcholines, phosphatidylethanolamines and their ether-linked subclass plasmenyl-phosphatidylethanolamines, phosphatidylglycerols, the sterol lipid subclass cholesteryl esters, and free fatty acids (see Supplementary Table S2 for lipid summary statistics). Overall, compared to females, males showed a more diffuse lipid profile, with no clear pattern in TGs and a broader set of age-related changes across lipid classes.

**FIGURE 4:**
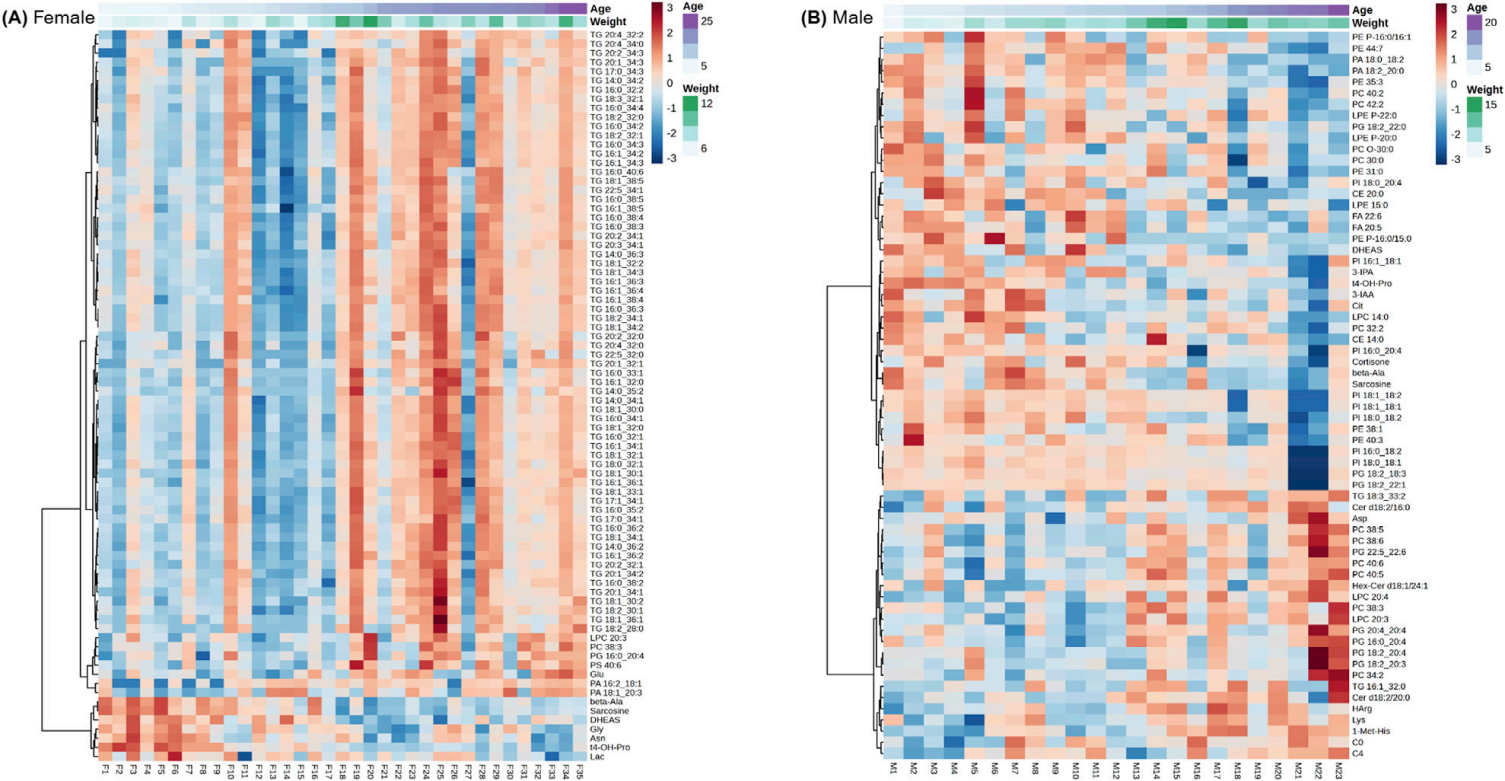
Heatmaps of age-associated metabolites identified by linear modeling for females only **(A)**, and males only **(B)**. Rows represent metabolites and columns represent individual animals ordered by age clustering. Metabolites concentrations are scaled and colored by relative concentrations (Z-Score) with signifying a high and blue a low concentration. Age and weight metadata are annotated on the upper x-axis. A Benjamini–Hochberg adjusted p FDR < 0.05 was considered significant.

To further examine individual variability in metabolite profiles across age by sex while accounting for weight, heatmaps were generated using the top age-associated metabolites identified by linear modeling (Figure 4). In females, 80 metabolites were included in the heatmap, and clustering showed that all the metabolites that were positively associated with age were 66 TG species (Figure 4A). Additional lipids in the glycerophospholipid class, including lysophosphatidylcholines, phosphatidylcholines, phosphatidylglycerols, phosphatidylserines, and phosphatidic acids, also increased with female age, while several amino acids and related metabolites, as well as DHEAS, decreased. These associations were independent of body weight, supporting the interpretation that these metabolic shifts reflect intrinsic aging processes rather than differences in somatic features such as muscle mass or fat stores. Notably, individual variation between females was evident. For example, subject F27, an 18.5-year-old female, exhibited a metabolite profile similar to that of younger individuals, deviating from the overall age trend.

In males, 50 metabolites were selected for heatmap visualization (Figure 4B). TGs were not among the top age-associated metabolites in this male cohort, but several lysophosphatidylcholines, phosphatidylcholines, phosphatidic acids, and cholesteryl esters were prominent, with several presenting either increases or decreases with age. A subset of amino acids and related metabolites also exhibited age-associated changes. Compared to females, the male cohort exhibited greater inter-individual variability and a more diffuse pattern of metabolic aging, with no single lipid class dominating the profile.

### 3.3 Sex-stratified GAM analysis of age-correlated metabolites

To further investigate the nonlinear patterns and sex differences observed in the correlation and heatmap analyses, GAMs were applied to age-associated metabolites. P-values for the smooth terms indicate whether age significantly predicted metabolite concentration within each sex after accounting for smoothing penalties, while F-tests assessed sex-by-age interactions. See Table 1 for a summary of all changed metabolites in males and females.

**TABLE 1:** Age-associated changes in serum metabolites and lipid species stratified by sex. Metabolites were grouped based on whether they exhibited significant associations with age in both sexes, in females only, or in males only. Lipid classes reflect broader metabolite families; individual lipid species vary in direction and significance between sexes.

Changed in females and males	Changed in females	Changed in males
Trans-4-Hydroxyproline (t4-OH-Pro)	Methionine	1-Methylhistidine (1-Met-His)
Beta-Alanine	Threonine	Lysophosphatidylethanolamines
Sarcosine	Tyrosine	Phosphatidylethanolamines
Dehydroepiandrosterone sulfate (DHEAS)	Glycine	Cholesteryl esters
Lysophosphatidylcholines	Asparagine	Free fatty acids
Phosphatidylcholines	Glutamate	Phosphatidylinositols
Phosphatidic acids	Hexose	Ether-linked phosphatidylethanolamines
	Indole-3-propionic acid (3-IPA)	Ceramides
	Triglycerides (TGs)	
	Phosphatidylglycerols	
	Phosphatidyl serines	

**FIGURE 5:**
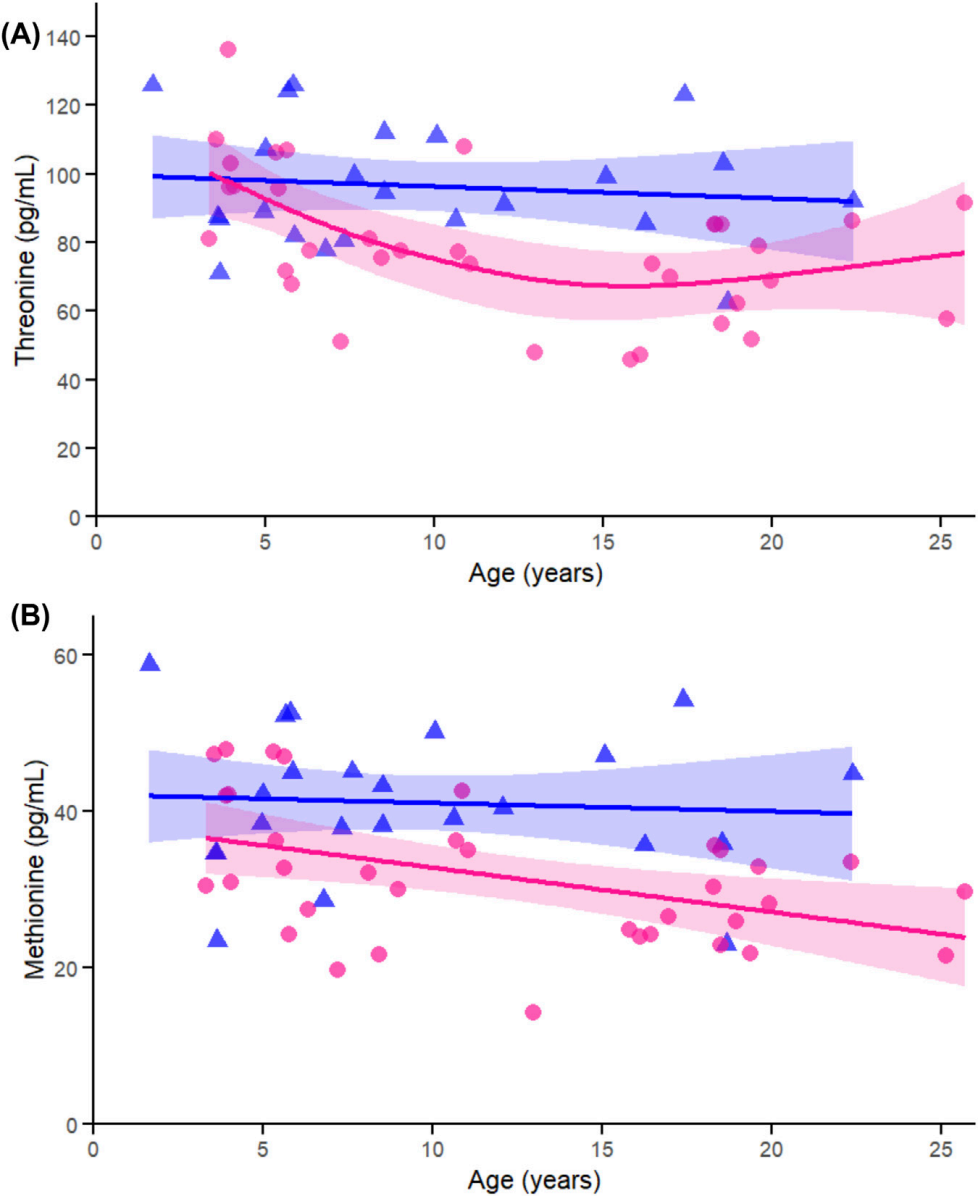
Age-related generalized additive models (GAMs) of selected significant (FDR < 0.05) essential proteinogenic amino acids in female (pink circles, red curve) and male (blue triangles, blue curve) rhesus macaques. Serum concentrations (pg/mL) are plotted against age (years), with fitted sex-specific regression lines. Panels show: **(A)** Methionine (Met). **(B)** Threonine (Thr). A p-Value < 0.05 was considered significant.

Among the proteinogenic essential amino acids, threonine (Figure 5A) exhibited a significant effect of age in females (p < 0.005) and a significant sex-by-age interaction (p = 0.032), consistent with earlier sex-stratified correlations. Methionine (Figure 5B) also showed a significant age-related association in females (p = 0.021) but no interaction, reinforcing the female-driven patterns observed in the initial analyses.

**FIGURE 6:**
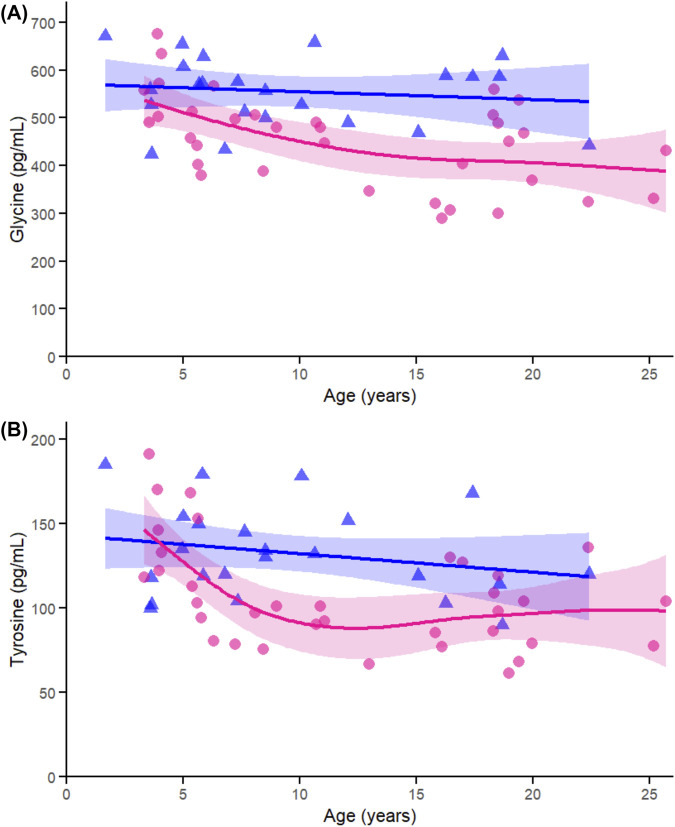
Age-related generalized additive models (GAMs) of selected significant (FDR < 0.05) conditionally essential proteinogenic amino acids in female (pink circles, red curve) and male (blue triangles, blue curve) rhesus macaques. Serum concentrations (pg/mL) are plotted against age (years), with fitted sex-specific regression lines. Panels show: **(A)** Tyrosine (Tyr), **(B)** Glycine (Gly). A p-Value < 0.05 was considered significant.

Within the conditionally essential amino acids, glycine (Figure 6A) was significantly associated with age in females (p < 0.005), not in males (p = 0.569), and did not show a significant interaction (p = 0.135). Tyrosine (Figure 6B) similarly showed an age effect in females (p < 0.005), not in males (p = 0.250), and exhibited a significant sex-by-age interaction (p = 0.013). These results align with the sex-specific decline in amino acids observed in the correlation and heatmap data.

**FIGURE 7:**
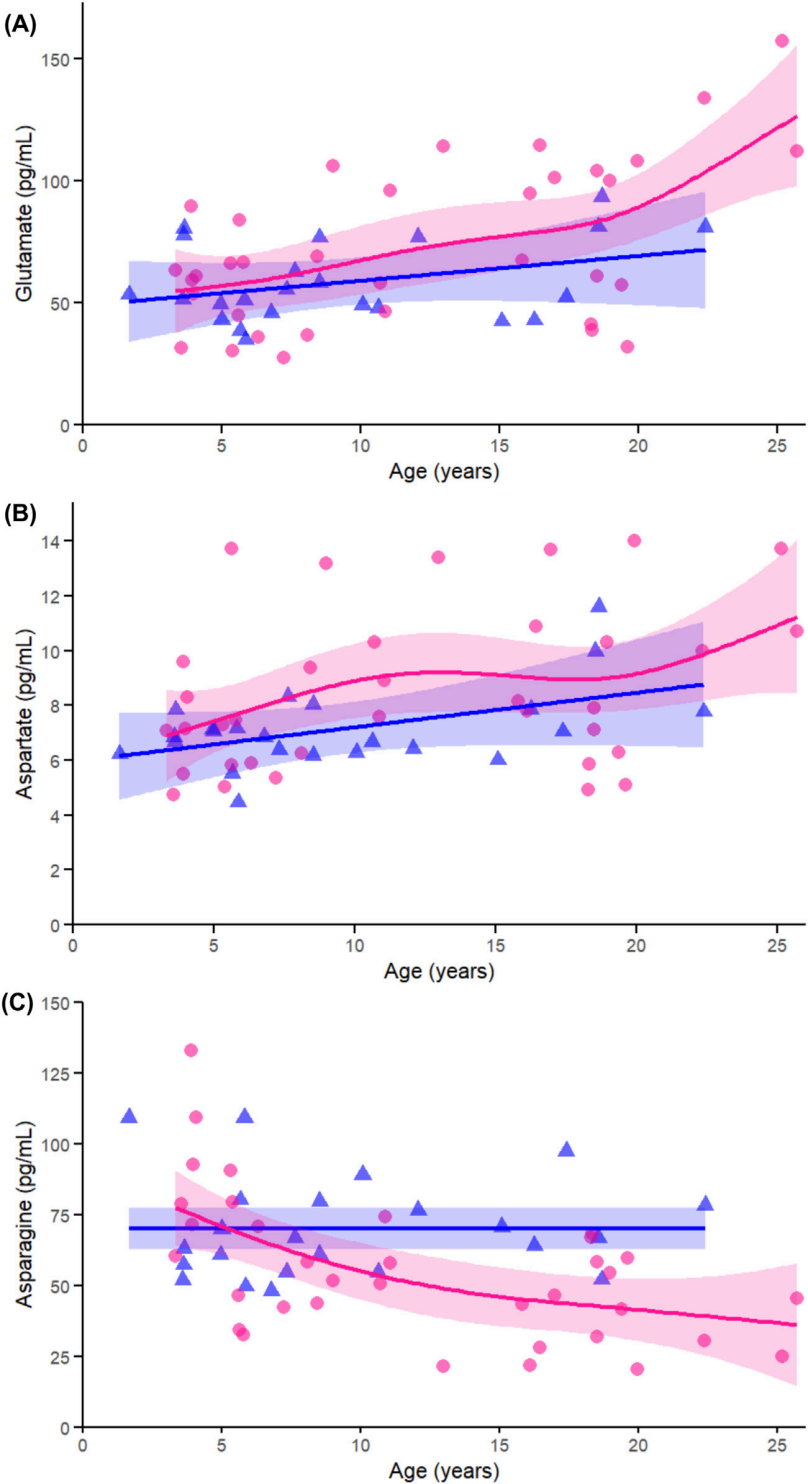
Age-related GAMs of selected significant (FDR < 0.05) essential proteinogenic amino acids in female (pink circles, red curve) and male (blue triangles, blue curve) rhesus macaques. Serum concentrations (pg/mL) are plotted against age (years), with fitted sex-specific regression lines. Panels show: **(A)** Glutamate (Glu), **(B)** Aspartate (Asp), **(C)** Asparagine (Asn). A p-Value < 0.05 was considered significant.

Across the non-essential amino acids, glutamate (Figure 7A) showed a significant age-related change in females (p < 0.005) but not in males (p = 0.241), with no significant interaction (p = 0.266), again supporting the female-specific nature of this trajectory. Aspartate (Figure 7B) did not show significant associations with age in either sex and showed no interaction (p = 0.343), consistent with weaker patterns shown in the correlation data. In contrast, asparagine (Figure 7C) was significantly associated with age in females (p < 0.005), not in males (p = 0.939), and had a moderate interaction (p = 0.023), confirming both the sex difference and its relevance to the interaction analysis.

**FIGURE 8:**
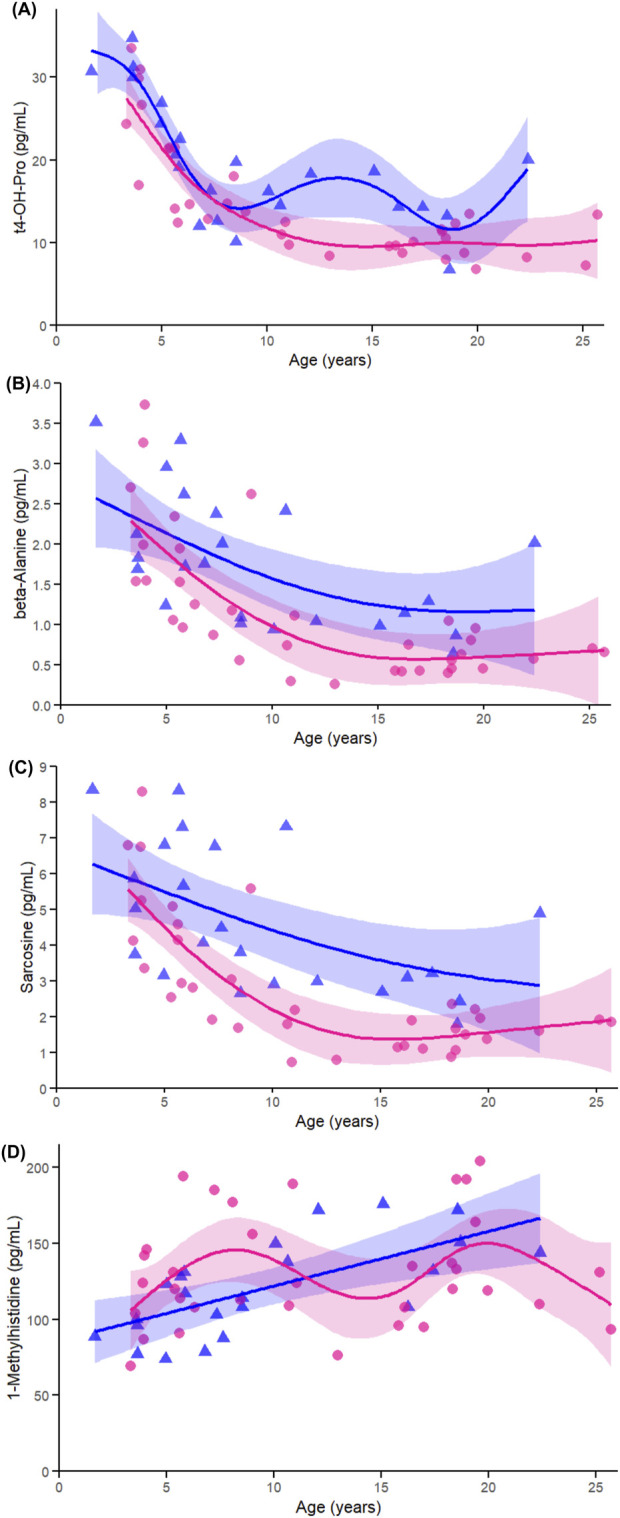
Age-related GAMs of selected significant (FDR < 0.05) non-proteinogenic amino acids and related metabolites in female (pink circles, red curve) and male (blue triangles, blue curve) rhesus macaques. Serum concentrations (pg/mL) are plotted against age (years), with fitted sex-specific regression lines. Panels show: **(A)** trans-4-Hydroxyproline (t4-OH-pro), **(B)** β-alanine (beta-ala), **(C)** Sarcosine, and **(D)** 1-methylhistidine. A p-Value < 0.05 was considered significant.

Among non-proteinogenic amino acids and related metabolites, t4-OH-Pro (Figure 8A), showed significant associations with age in both females and males (both p < 0.005), with a trend toward a sex-by-age interaction (p = 0.063). This finding is consistent with the strong cross-sex correlations observed earlier and its prominence among the top-ranked age-associated metabolites. Beta-alanine (Figure 8B) and sarcosine (Figure 8C) also showed significant age-related declines in both sexes (all p < 0.005), with no evidence of sex-by-age interaction (beta-alanine p = 0.625; sarcosine p = 0.570), reinforcing their shared age-related trajectories identified in both correlation and heatmap analyses. In contrast, 1-Met-His (Figure 8D) exhibited a significant age-related increase in males (p < 0.005), but not in females (p = 0.072), and a significant sex-by-age interaction (p < 0.005), in agreement with earlier sex-stratified results highlighting its male-specific pattern.

**FIGURE 9:**
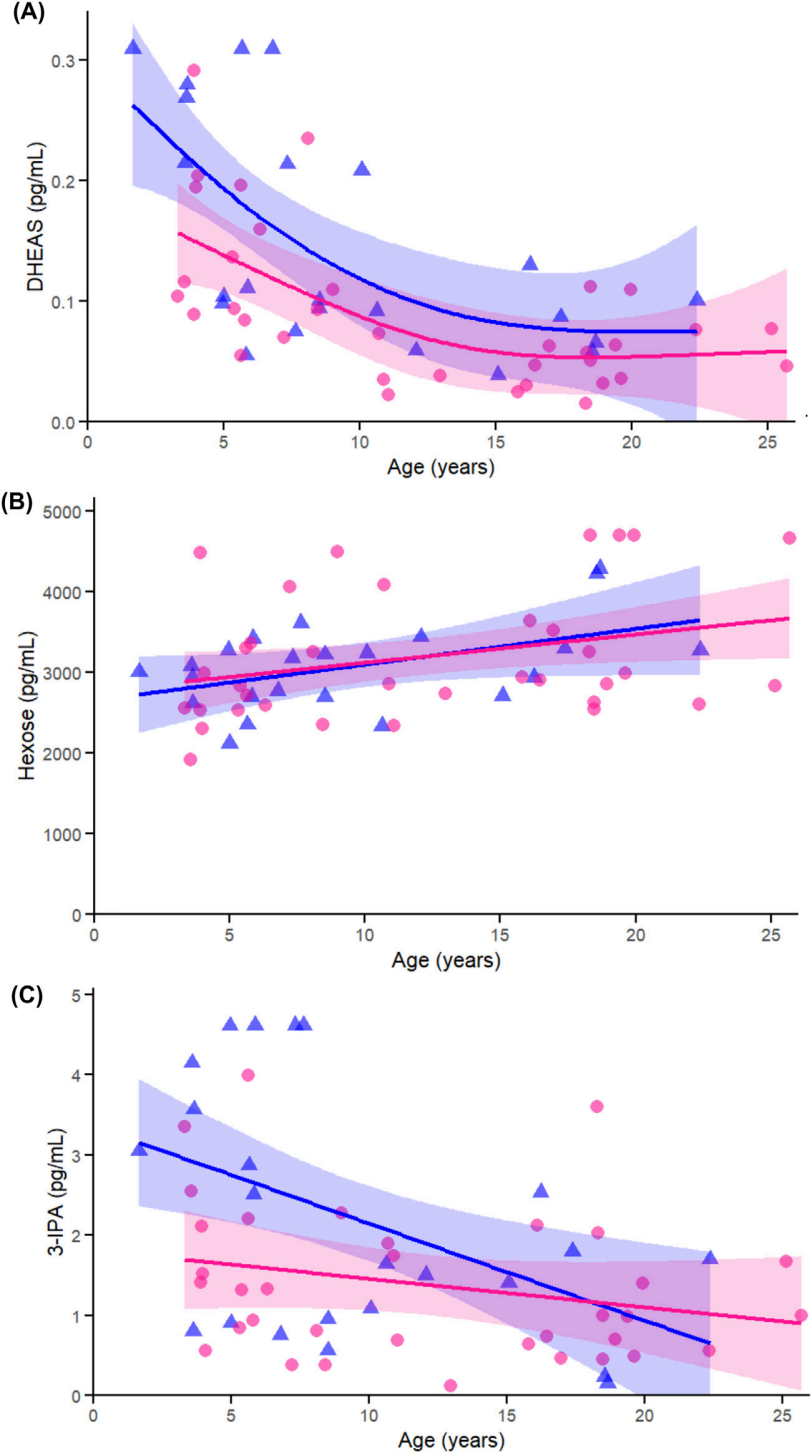
Age-related GAMs of selected significant (FDR <0.05) metabolites in female (pink circles, red curve) and male (blue triangles, blue curve) rhesus macaques. Serum concentrations (pg/mL) are plotted against age (years), with fitted sex-specific regression lines. **(A)** dehydroepiandrosterone sulfate (DHEAS) **(B)** Hexose **(C)** 3-Indolepropionic acid (3-IPA). A p-Value < 0.05 was considered significant.

DHEAS (Figure 9A) declined with age in both females (p = 0.038) and males (p < 0.005), with no evidence of a sex-by-age interaction (p = 0.930). This pattern aligns with earlier correlation and heatmap results demonstrating a broad and consistent age-associated decline across sexes. Hexose (Figure 9B) showed a modest increase with age in females (p = 0.022) and a trend in males (p = 0.097), with no interaction (p = 0.933), reinforcing its role as an energy-related metabolite that may reflect age-related changes in glucose regulation, particularly in females. 3-IPA (Figure 9C) was significantly associated with age in females (p = 0.005), not in males (p = 0.205), and showed no interaction (p = 0.098), consistent with earlier correlation findings and suggesting a possible female-specific decline in this gut microbial metabolite.

## 4 Discussion

The results of this study revealed specific age-related changes in metabolites involved in hormone metabolism (e.g., DHEAS), energy metabolism (e.g., hexose), and amino acid biosynthesis and catabolism (e.g., beta-alanine, sarcosine, t4-OH-pro) in male and female rhesus macaques. These findings are broadly consistent with prior aging studies in humans, reinforcing the value of the rhesus macaque as a translational model for studying the metabolic signatures of aging. Given the differential profiles of aging in between human men and women, a major focus of this study was the determination of metabolites that differed by age between male and female macaques. Sex emerged as a key modifier of age-related metabolic trajectories in serum with a variety of lipids, amino acids and related compounds, and gut microbial species altered between sexes. Females demonstrated a profound increase in serum TGs, amino acids, and other small molecules. In contrast, males exhibited a far more heterogenous profile with changes in a number of lipid types, but, interestingly, no TGs were significantly affected. Male rhesus also exhibited altered levels of amino acids and related metabolites, hormones, gut microbial, and energy metabolism metabolites.

### 4.1 Sex influences lipid metabolite profile across the lifespan

Lipids play diverse physiological roles including forming cell membranes, cell signaling effects, and the storage and mobilization of energy. Lipids have been shown to be potent regulators of longevity in longer lived species (Johnson and Stolzing, 2019), and, given the increasing ease of measurement (i.e., targeted metabolomics) allowing for identification of individual species vs. composite measures, they are emerging as powerful biomarkers of aging (Han and Ye, 2021).

The lipid profile of the rhesus in our study changed with age, but in dissimilar profiles based on sex, while controlling for body weight. TGs are the most common type of lipid in the blood, with their major role as energy storage in in adipose cells (Scordo and Pickett, 2017). Their structure is 3 fatty acids bound to glycerol and can be synthesized in the body as well as consumed in food (Scordo and Pickett, 2017). It is well established that there is a positive correlation between circulating levels of TGs and age in human and rhesus macaques, which has mostly been attributed to a delay in postprandial clearance with increasing age (Spitler and Davies, 2020). This increase in TGs is associated with morbidity including heart disease and diabetes (Han and Ye, 2021).

Sex differences in blood levels of TGs have been previously demonstrated in female humans (Jensen et al., 1990; Tabassum et al., 2023) and rhesus macaques (Ramsey et al., 2000). In the current study, the age-related elevation in 66 of the 177 TGs measured in the present study occurred independently of body weight or diet composition, indicating a conserved, intrinsic metabolic shift during aging. An explanation for the elevation in TGs with age in females may lie in endocrine regulation of lipid metabolism: estrogens have been shown to suppress hepatic TG production and modulate lipid storage and mobilization across tissues (Mauvais-Jarvis, 2015). As estrogen levels decline with age, as occurs in female humans and macaques (Kohama and Urbanski, 2024), these regulatory mechanisms may become dysregulated, leading to increased circulating TGs.

The age-related shifts in other lipid classes, including phosphatidylethanolamines, phosphatidylinositols, phosphatidylcholines, and ceramides suggest broader changes in cell membrane composition, lipid signaling, and metabolic regulation with advancing age. Phosphatidylethanolamines and phosphatidylinositols are key components of cellular membranes and play critical roles in vesicle trafficking, autophagy, and intracellular signaling (Calzada et al., 2016; Van Der Veen et al., 2019). Phosphatidylcholines, another membrane lipid class, are also involved in lipoprotein assembly and lipid transport (Kent, 2005). Ceramides, bioactive sphingolipids implicated in inflammation, insulin resistance, and cellular senescence, have been linked to age-related cellular dysfunction (Hulbert, 2005).

Collectively, these findings point to a complex reorganization of lipid metabolism with age. The parallels between human and non-human primate lipidomic aging profiles support the translational value of rhesus macaques as a model for studying metabolic aging. Importantly, the findings underscore the need to examine lipids in a nuanced class-specific manner to reveal physiologically meaningful patterns and to identify candidate biomarkers of aging that would otherwise be obscured by aggregate lipid analysis.

### 4.2 Proteinogeneic amino acids are affected by age and sex

To our knowledge, the current study is the first to demonstrate age-related changes in proteinogenic amino acids in the rhesus macaque and that these changes occur in a sex-specific manner. Analysis of the metabolomic data with both sexes combined identified that the proteinogenic amino acids threonine, methionine, glycine, tyrosine, and asparagine decreased with age, while glutamate and aspartate increased. GAM analysis showed that all age-related changes in proteinogenic amino acids were significant exclusively in females, with no significant trajectories observed in males. Age by sex interactions were found for threonine, tyrosine, and asparagine, indicating sex-specific divergence in age-related amino acid regulation. These effects may reflect both biological differences and the greater representation of older females in the dataset.

The age-related patterns in levels of proteinogenic amino acids observed in this study are consistent with established alterations in amino acid utilization and turnover observed in other aging organisms (Deutz et al., 2024). These amino acids play a role in key metabolic pathways that regulate redox balance, protein synthesis, immune function, neurotransmitter biosynthesis, and autophagy. The significant age-related increase in hexose levels in female and male rhesus is consistent with impaired glucose utilization and altered redox control. This pattern may further indicate reduced nicotinamide adenine dinucleotide phosphate (NADPH) availability for glutathione regeneration, reinforcing the coordinated decline in amino acid and energy metabolism with age. In human and animal models, glycine has been implicated in pro-longevity pathways through its role in glutathione synthesis, methionine load reduction, and the stimulation of autophagy via conversion to sarcosine (Johnson & Cuellar, 2023), with supplementation studies supporting its ability to reduce inflammation and enhance metabolic health (Soh et al., 2023; Deutz et al., 2024). In this study, however, sarcosine significantly declined with age (discussed below) therefore together, these findings may reflect a reduction in glycine N-methyltransferase (GNMT) activity, limited methyl donor availability, or a broader decline in methylation-driven regulatory processes with age.

Changes in tyrosine, a precursor of dopamine, norepinephrine, and epinephrine, may indicate declining catecholamine biosynthesis with age (Fernstrom and Fernstrom, 2007; Litwack, 2018), which is consistent with reduced tyrosine availability and altered amino acid metabolism in older humans (Pitkänen et al., 2003).

In contrast to the amino acids that declined with age, glutamate and aspartate levels increased, but as stated above, significant only in female rhesus based on GAM analysis. Although this pattern diverges from some human studies (Pitkänen et al., 2003), it is consistent with prior findings in macaque brain tissue and may reflect species-specific or tissue-specific regulation of excitatory neurotransmitters (Quintero et al., 2007). Elevated circulating glutamate and aspartate may result from enhanced proteolysis, altered mitochondrial flux, or imbalances in nitrogen and redox metabolism, each of which would be expected to shift with age.

Accumulated evidence indicates that environmental factors, like diet, can affect metabolite profiles. For example, methionine restriction in aging rats and human extends lifespan and delays metabolic and immune decline (Miller et al., 2005; Kitada et al., 2021), likely through effects on glutathione and polyamine synthesis and through epigenetic regulation via S-adenosylmethionine (Lauinger and Kaiser, 2021). Interestingly, amino acids like asparagine show variable trajectories with age across human studies (Pitkänen et al., 2003; Kouchiwa et al., 2012). As our macaques were on controlled housing conditions and fed a standardized diet, the identified patterns were most probably driven by internal shifts in metabolism, such as reduced hepatic gluconeogenesis or altered skeletal muscle flux, rather than by dietary intake. In that regard, threonine requirements usually remain stable in older adults (Tontisirin et al., 1974), yet decreased intake or metabolic efficiency may impair nitrogen balance and protein retention (Tang et al., 2021).

The identified age-related patterns in the levels of proteinogenic amino acids support the concept that multiple components of amino acid metabolism, including neurotransmission and energy-linked shuttles, are affected during aging and stress the importance of considering sex-specific trajectories.

### 4.3 Non-proteinogenic amino acids are preferentially impacted by age

Several non-proteinogenic amino acids, including sarcosine, beta-alanine, and t4-OH-Pro, declined significantly with age in both sexes. Although not incorporated into proteins, these metabolites contribute to key physiological processes such as methylation balance, oxidative stress defense, and tissue turnover. These functions overlap with those of proteinogenic amino acids that were also found to change with age.

Sarcosine is involved in methionine, glycine, and folate metabolism and supports methylation, mitochondrial function, and creatine synthesis. Dietary restriction in rats restores sarcosine levels along with increased autophagy, implicating it in pathways that support cellular maintenance and longevity. The decline of sarcosine with age in macaques parallels findings in aging humans (Walters et al., 2018).

Beta-alanine, a precursor to carnosine, contributes to muscle pH buffering and antioxidant defense (Derave et al., 2010). Its age-related decline may reflect reduced muscular resilience, which is consistent with studies showing that supplementation improves carnosine levels and physical performance in humans (Harris et al., 2006).

T4-OH-Pro, a product of collagen degradation, indicates connective tissue remodeling and plays a role in stabilizing the collagen triple helix (Mizuno et al., 2003). Its decline in aging humans is associated with reduced skin elasticity and joint integrity (Varani et al., 2006), and a recent study in aging mice identified consistent reductions across 11 of 12 organs (Pilley et al., 2024). Our finding of decreased age-related t4-OH-pro in rhesus macaques extend this pattern to circulating levels in rhesus macaques, supporting its potential as a biomarker of age-related connective tissue decline.

In males, 1-Met-His was the only non-proteinogenic amino acid to increase significantly with age. This metabolite is released alongside beta-alanine during the degradation of anserine, a dipeptide abundant in skeletal muscle (Aranibar et al., 2011). Interestingly, while beta-alanine declined with age in both sexes, the male-specific increase in 1-Met-His may reflect altered anserine turnover or skeletal muscle dynamics with age. Anserine has antioxidant properties (Wu et al., 2020) and has been shown to improve cognitive outcomes in APOE4+ mice (Caruso et al., 2021). Additionally, 1-Met-His has been shown to mediate the association between age and red blood cell distribution width (RDW), a clinical marker linked to adverse outcomes in aging humans (Tian et al., 2023). Together, these findings support a potential role for 1-Met-His as a sex-specific marker of age-related physiological shifts and highlight broader alterations in muscle-related metabolic processes during aging.

The decline of these non-proteinogenic amino acids complements the reductions observed in proteinogenic amino acids and points to a coordinated shift in metabolic pathways that support tissue maintenance, redox regulation, and energy metabolism during aging.

### 4.4 Changes in energy, DHEA, and gut microbial metabolites contribute to sex-related differential patterns across the lifespan

Serum hexose levels increased modestly with age in female rhesus and there was a trend towards a similar finding in males, consistent with impaired glucose utilization and altered redox regulation. This pattern complements the observed decline in amino acids involved in antioxidant defense, methylation, and energy metabolism, suggesting broader disruption in nutrient handling with age. Glucose, the primary circulating hexose, is essential for ATP production and is regulated by insulin in both humans and rhesus macaques (Hansen and Bodkin, 1986). In humans, fasting plasma glucose increases progressively with age and is associated with metabolic syndrome and immune dysregulation (Chia et al., 2018; Swarup et al., 2025; Xu et al., 2025). Similarly, aging in rhesus is associated with hyperinsulinemia, reduced glucose clearance (Gresl et al., 2001), elevated fasting glucose, and diminished insulin secretion (Zhu et al., 2023). The aforementioned reports, combined with the trajectory observed in females in this study suggest that age-related changes in glucose metabolism of primates may emerge earlier or more prominently in females, potentially linked to sex differences in systemic energy demands or tissue-specific insulin sensitivity.

Serum DHEAS levels declined with age in both sexes, with females consistently exhibiting lower concentrations than males. As the most abundant circulating steroid in humans and nonhuman primates, DHEAS acts as a precursor to DHEA and downstream androgens and estrogens (Samaras et al., 2013; Mohamad et al., 2024). While neither of these hormones were measured in this study, lower DHEAS levels align with decreased estrogen regulation and higher TGs in females. The sex difference and age-related decline in DHEAS levels have been previously reported in rhesus monkeys (Lane et al., 1997) and humans (Ravaglia et al., 2002). In older adults, lower DHEAS has been associated with reduced muscle mass and strength (Valenti et al., 2004) and better cognitive performance in women with higher levels (Davis et al., 2008), although supplementation trials have shown no benefit on insulin sensitivity, body composition, or physical performance (Nair et al., 2006).

Serum levels of 3-IPA, a gut microbial metabolite derived from dietary tryptophan, declined with age, which was only significant in female rhesus based on GAM analysis. In humans, 3-IPA levels have been reported to be approximately ten times lower in older adults compared to younger individuals (Kim et al., 2023), indicating a substantial age-related decline in accordance with the data from this study. 3-IPA has demonstrated neuroprotective and anti-inflammatory properties, including associations with higher circulating brain-derived neurotrophic factor in older individuals receiving probiotics (Kim et al., 2023). Mechanistically, 3-IPA reduces TNF-α production in activated microglia and promotes expression of BDNF and nerve growth factor in neurons, supporting its role in maintaining neuronal health (Kim et al., 2023). The observed decline of 3-IPA in this study may reflect changes in microbial metabolism with age and could contribute to neuroinflammatory processes and cognitive decline, highlighting a potential link between the gut microbiome, aging, and brain health.

### 4.5 Final thoughts and conclusions

Overall, this nonhuman primate study underscores the utility of targeted metabolomics as a tool for investigating multiple facets of aging biology and translational research. With a relatively simple sample preparation, this approach enabled measurement of over 600 metabolites, providing insight into physiological pathways underlying sex-specific aging in 58 rhesus macaques. The metabolite profiles identified individuals who deviated from population-level aging trends, offering potential markers of biological age as distinct from chronological age and for targeted precision medicine approaches. The generation of absolute concentration data supports the future development of single-analyte assays, reducing reliance on comprehensive platforms and facilitating broader translational applications in aging research and clinical monitoring.

Aging is the most significant risk factor for neurodegenerative diseases (Hou et al., 2019) and several of the age-associated metabolites identified in this study have previously been linked to such conditions (e.g., Sorwell and Urbanski, 2010; Höllig et al., 2015; Wyss-Coray, 2016; Tanas et al., 2022; Kim et al., 2023; Ostfeld et al., 2023; Xiao et al., 2025). This highlights that normal aging in rhesus macaques involves metabolic alterations in pathways that are highly relevant to neurodegenerative processes, supporting the use of this model for translational investigation into early metabolic risk markers of neurological decline.

Three main limitations can be identified in this report. First, the oldest male was aged 22 years while the oldest female was 25 years old. The age imbalance may have contributed to the observed sex differences. To overcome this issue, age was modeled as a continuous variable, and consistent patterns support the validity of the findings. We also recognize that although rhesus macaques may be considered old at >22 years of age, individuals can live up to age 41 (Simmons, 2016), and the absence of older animals may limit generalizability to late-life stages. Second, the cross-sectional design does not allow inference of intra-individual change over time. Although age-related trends were observed, longitudinal data are needed to confirm whether these patterns reflect within-subject aging rather than cohort effects. Lastly, while the overall sample size was strong for a nonhuman primate study (n = 58), the sex distribution was skewed toward females (n = 35) over males n = 23) due to availability for blood sampling. This difference potentially reduced the power to detect male-specific trajectories and limiting interpretation of direct sex comparisons.

In conclusion, this study provides the first comprehensive characterization of sex-dependent serum metabolomic aging patterns in rhesus macaques. We identified distinct metabolic trajectories in males and females, including divergent lipid profiles and age-related shifts in amino acid metabolism that were significant only in females. These findings emphasize the importance of incorporating sex as a biological variable in aging research and support the use of rhesus macaques as a translational model for identifying metabolic biomarkers of aging. Continued investigation of these signatures may inform sex-specific approaches to monitoring and mitigating age-related physiological decline.

## Data Availability

The original contributions presented in the study are publicly available. This data can be found here: http://datadryad.org/share/0K5D5P9q6rJFxJOxErM_qPx4N1iNT_d--nDYDf5glW4.
