# Supplementary material for: Comparative analysis of sex-dependent serum metabolomic patterns across the lifespan of rhesus macaques
Source: Front Genet. 2025 Aug 25;16:1655325. doi: 10.3389/fgene.2025.1655325 (PMC12414774; doi:10.3389/fgene.2025.1655325)
Supplement: Supplementary file 1 [file Supplementaryfile1.docx]

**Table S1:** Demographic information for each male (M) rhesus macaque included in the study population.

| **M**  **ID** | **Age (years)** | **Weight (kg)** |
| --- | --- | --- |
| **M1** | 1.66 | 3.2 |
| **M2** | 3.59 | 5.84 |
| **M3** | 3.63 | 5.16 |
| **M4** | 3.65 | 4.37 |
| **M5** | 4.96 | 9.72 |
| **M6** | 5 | 6.38 |
| **M7** | 5.66 | 9.9 |
| **M8** | 5.82 | 10.15 |
| **M9** | 5.87 | 11.3 |
| **M10** | 6.79 | 8.38 |
| **M11** | 7.33 | 9.4 |
| **M12** | 7.63 | 9.19 |
| **M13** | 8.52 | 12.74 |
| **M14** | 8.52 | 17.45 |
| **M15** | 10.07 | 19.75 |
| **M16** | 10.63 | 10.26 |
| **M17** | 12.07 | 15.17 |
| **M18** | 15.08 | 18.77 |
| **M19** | 16.25 | 9.87 |
| **M20** | 17.39 | 13.48 |
| **M21** | 18.55 | 11.82 |
| **M22** | 18.67 | 11.26 |
| **M23** | 22.39 | 13.46 |

**Table S2:** Demographic information for each female (F) rhesus macaque included in the study population.

| **F**  **ID** | **Age (years)** | **Weight (kg)** | **Parity** |
| --- | --- | --- | --- |
| **F1** | 3.33 | 4.64 | 0 |
| **F2** | 3.56 | 5.26 | 0 |
| **F3** | 3.92 | 5.74 | 0 |
| **F4** | 3.93 | 5.5 | 0 |
| **F5** | 3.98 | 5.1 | 1 |
| **F6** | 4.07 | 4.61 | 0 |
| **F7** | 5.31 | 7.2 | 0 |
| **F8** | 5.39 | 5.08 | 0 |
| **F9** | 5.62 | 5.58 | 3 |
| **F10** | 5.63 | 7.7 | 0 |
| **F11** | 5.79 | 5.45 | 1 |
| **F12** | 6.33 | 4.96 | 1 |
| **F13** | 7.22 | 8.37 | 0 |
| **F14** | 8.1 | 7.15 | 0 |
| **F15** | 8.44 | 4.85 | 0 |
| **F16** | 9 | 7.62 | 0 |
| **F17** | 10.7 | 8.12 | 1 |
| **F18** | 10.89 | 12.6 | 0 |
| **F19** | 11.06 | 9.9 | 0 |
| **F20** | 12.96 | 13.66 | 0 |
| **F21** | 15.82 | 9.64 | 4 |
| **F22** | 16.11 | 6 | 2 |
| **F23** | 16.45 | 7.86 | 2 |
| **F24** | 16.97 | 10.39 | 4 |
| **F25** | 18.3 | 7.36 | 11 |
| **F26** | 18.34 | 6.44 | 9 |
| **F27** | 18.49 | 9.25 | 6 |
| **F28** | 18.49 | 7.06 | 6 |
| **F29** | 18.95 | 10.96 | 1 |
| **F30** | 19.39 | 5.95 | 8 |
| **F31** | 19.62 | 9.17 | 7 |
| **F32** | 19.95 | 8.41 | 2 |
| **F33** | 22.36 | 7.16 | 9 |
| **F34** | 25.15 | 12.13 | 9 |
| **F35** | 25.71 | 8.06 | 10 |

**Table S3:** Results of correlation analysis between serum metabolites and age in rhesus macaques. For each metabolite, Pearson correlation coefficients (r), t-values, nominal p-values, and false discovery rate (FDR)–adjusted p-values are reported. Negative correlation coefficients indicate decreasing metabolite concentrations with age, while positive coefficients indicate age-associated increases. Significance is denoted as follows: * p FDR < 0.05, ** p FDR < 0.01, and *** p FDR < 0.005.

| **Metabolite** | **Correlation coefficient (r)** | **t-value** | **p-value** | **FDR (False discovery rate)** |
| --- | --- | --- | --- | --- |
| **t4-OH-Pro** | -0.72783 | -7.9424 | 9.619e-11 | 5.3482e-08*** |
| **beta-Ala** | -0.65082 | -6.4148 | 3.1888e-08 | 7.2974e-06*** |
| **Sarcosine** | -0.64754 | -6.359 | 3.9374e-08 | 7.2974e-06*** |
| **DHEAS** | -0.57128 | -5.2086 | 2.8237e-06 | 0.00039249*** |
| **LPC 20:3** | 0.50751 | 4.4077 | 4.7793e-05 | 0.0053146** |
| **PA 18:2_20:0** | -0.50114 | -4.3336 | 6.1539e-05 | 0.0057026** |
| **PC 38:3** | 0.48424 | 4.1418 | 0.00011739 | 0.0093244** |
| **PA 18:0_18:2** | -0.48028 | -4.0976 | 0.00013594 | 0.0094481** |
| **PG 16:0_20:4** | 0.47352 | 4.0231 | 0.00017392 | 0.010744* |
| **TG 16:1_32:0** | 0.47005 | 3.9852 | 0.00019692 | 0.010949* |
| **FA 20:5** | -0.46486 | -3.929 | 0.00023659 | 0.011959* |
| **PC 38:6** | 0.45863 | 3.8622 | 0.00029384 | 0.013615* |
| **PG 22:5_22:6** | 0.44774 | 3.7472 | 0.00042481 | 0.018169* |
| **Gly** | -0.44335 | -3.7013 | 0.00049128 | 0.018248* |
| **Asn** | -0.44117 | -3.6787 | 0.00052758 | 0.018248* |
| **Glu** | 0.43975 | 3.6641 | 0.0005525 | 0.018248* |
| **TG 20:2_32:1** | 0.43873 | 3.6536 | 0.00057106 | 0.018248* |
| **PC 40:6** | 0.43768 | 3.6428 | 0.00059075 | 0.018248* |
| **PI 16:0_20:0** | 0.43158 | 3.5802 | 0.00071825 | 0.020802* |
| **TG 16:1_34:1** | 0.43009 | 3.5651 | 0.0007528 | 0.020802* |
| **TG 22:5_32:0** | 0.42873 | 3.5513 | 0.0007857 | 0.020802* |
| **ADMA** | -0.42617 | -3.5253 | 0.0008514 | 0.020989* |
| **PC 38:5** | 0.42554 | 3.5189 | 0.00086824 | 0.020989* |
| **PC 40:5** | 0.41958 | 3.459 | 0.0010434 | 0.024171* |
| **PE 35:3** | -0.41331 | -3.3966 | 0.0012614 | 0.027182* |
| **TG 16:0_33:1** | 0.4123 | 3.3866 | 0.0012998 | 0.027182* |
| **Tyr** | -0.41023 | -3.3662 | 0.0013825 | 0.027182* |
| **TG 18:1_32:0** | 0.40952 | 3.3592 | 0.0014119 | 0.027182* |
| **PG 18:2_20:3** | 0.40828 | 3.3469 | 0.0014649 | 0.027182* |
| **TG 20:4_34:0** | 0.40824 | 3.3465 | 0.0014666 | 0.027182* |
| **PS 40:6** | 0.40451 | 3.31 | 0.0016361 | 0.029344* |
| **TG 16:0_34:1** | 0.40342 | 3.2993 | 0.001689 | 0.029346* |
| **TG 18:1_36:1** | 0.40203 | 3.2857 | 0.0017586 | 0.02963* |
| **TG 20:3_34:1** | 0.39857 | 3.2521 | 0.0019428 | 0.031771* |
| **Thr** | -0.39618 | -3.229 | 0.0020797 | 0.031892* |
| **TG 16:0_38:5** | 0.39542 | 3.2216 | 0.0021255 | 0.031892* |
| **TG 18:1_38:5** | 0.39206 | 3.1892 | 0.002337 | 0.031892* |
| **TG 16:1_34:2** | 0.39106 | 3.1797 | 0.002403 | 0.031892* |
| **PG 18:2_20:4** | 0.39048 | 3.1741 | 0.0024422 | 0.031892* |
| **TG 18:2_32:0** | 0.39 | 3.1694 | 0.0024757 | 0.031892* |
| **SDMA** | -0.38974 | -3.1669 | 0.0024939 | 0.031892* |
| **LPE 20:3** | 0.38906 | 3.1605 | 0.0025413 | 0.031892* |
| **TG 16:0_32:1** | 0.38866 | 3.1566 | 0.0025698 | 0.031892* |
| **PC O-38:3** | 0.38857 | 3.1557 | 0.0025764 | 0.031892* |
| **TG 20:1_32:1** | 0.3885 | 3.1551 | 0.0025812 | 0.031892* |
| **TG 16:0_34:2** | 0.38605 | 3.1317 | 0.0027622 | 0.031966* |
| **TG 22:4_32:0** | 0.38545 | 3.126 | 0.0028085 | 0.031966* |
| **PC 36:3** | 0.38539 | 3.1254 | 0.0028129 | 0.031966* |
| **TG 20:1_34:1** | 0.38533 | 3.1249 | 0.0028172 | 0.031966* |
| **TG 20:4_32:0** | 0.38307 | 3.1033 | 0.0029978 | 0.033336* |
| **PI 18:0_20:0** | 0.38 | 3.0743 | 0.0032587 | 0.035526* |
| **PG 20:4_20:4** | 0.37846 | 3.0597 | 0.0033967 | 0.03573* |
| **Asp** | 0.37836 | 3.0588 | 0.0034059 | 0.03573* |
| **Cer d18:1/20:0** | 0.37492 | 3.0264 | 0.0037345 | 0.038452* |
| **CE 20:3** | 0.37327 | 3.011 | 0.0039018 | 0.038993* |
| **TG 16:0_34:3** | 0.37182 | 2.9974 | 0.0040544 | 0.038993* |
| **PI 16:0_18:2** | -0.37173 | -2.9965 | 0.0040641 | 0.038993* |
| **TG 16:0_32:0** | 0.3717 | 2.9962 | 0.0040676 | 0.038993* |
| **Hexose** | 0.36858 | 2.9671 | 0.004415 | 0.041606* |
| **TG 20:2_32:0** | 0.36719 | 2.9542 | 0.0045774 | 0.042417* |
| **TG 16:0_40:6** | 0.36595 | 2.9426 | 0.0047276 | 0.042456* |
| **TG 20:1_34:3** | 0.36568 | 2.9401 | 0.0047607 | 0.042456* |
| **TG 16:1_34:3** | 0.36489 | 2.9328 | 0.0048589 | 0.042456* |
| **TG 18:1_34:1** | 0.36436 | 2.9279 | 0.004926 | 0.042456* |
| **PC 34:3** | 0.36407 | 2.9252 | 0.0049634 | 0.042456* |
| **PC O-40:3** | 0.36076 | 2.8946 | 0.0054019 | 0.045258* |
| **TG 16:0_36:2** | 0.35998 | 2.8874 | 0.005511 | 0.045258* |
| **PC 38:4** | 0.35891 | 2.8776 | 0.0056619 | 0.045258* |
| **TG 16:1_36:2** | 0.35882 | 2.8767 | 0.0056754 | 0.045258* |
| **TG 18:1_34:2** | 0.35866 | 2.8753 | 0.0056979 | 0.045258* |
| **PC 34:2** | 0.35793 | 2.8686 | 0.0058041 | 0.045452* |
| **TG 18:1_30:1** | 0.35688 | 2.8589 | 0.0059611 | 0.046033* |
| **TG 16:0_36:3** | 0.35406 | 2.8331 | 0.0063972 | 0.046188* |
| **PC O-34:3** | -0.35357 | -2.8285 | 0.0064769 | 0.046188* |
| **TG 16:0_38:4** | 0.35318 | 2.825 | 0.0065395 | 0.046188* |
| **TG 20:4_34:1** | 0.35316 | 2.8248 | 0.0065433 | 0.046188* |
| **PG 18:0_18:3** | 0.35315 | 2.8248 | 0.0065438 | 0.046188* |
| **TG 18:2_34:1** | 0.35311 | 2.8244 | 0.0065505 | 0.046188* |
| **TG 22:5_34:1** | 0.35304 | 2.8237 | 0.0065627 | 0.046188* |
| **Met** | -0.35092 | -2.8044 | 0.0069175 | 0.047919* |
| **TG 18:2_31:0** | 0.35055 | 2.801 | 0.006981 | 0.047919* |
| **3-IPA** | -0.34916 | -2.7884 | 0.007223 | 0.048305* |
| **TG 17:1_34:1** | 0.34901 | 2.787 | 0.0072507 | 0.048305* |
| **PI 18:0_18:1** | -0.34839 | -2.7813 | 0.0073621 | 0.048305* |
| **PG 16:0_18:3** | 0.34826 | 2.7802 | 0.0073847 | 0.048305* |
| **PG 18:2_22:4** | 0.34735 | 2.7719 | 0.0075524 | 0.048827* |
| **CerP d18:1/16:0** | 0.34639 | 2.7632 | 0.0077311 | 0.049408* |

**Table S4:** Results of sex-specific generalized additive model (GAM) results for age-related trajectories of selected serum metabolites in rhesus macaques. Estimated degrees of freedom (edf) and associated p-values are reported separately for males and females. The "Interaction pValue" column reflects the significance of the age-by-sex interaction term. “n.a” denotes models where interaction terms were not applicable due to identical smooths or convergence issues. Significance is denoted as follows * p < 0.05, ** p < 0.01, and *** p < 0.005

| **Metabolite** | **edf for males** | **edf for females** | **pValue for males** | **pValue for females** | **Interaction pValue** |
| --- | --- | --- | --- | --- | --- |
| **Threonine** | 1 | 2.408 | 0.5900 | < 0.005*** | 0.0320* |
| **Methionine** | 1 | 1.764 | 0.7273 | 0.0213* | 0.2361 |
| **Glycine** | 1 | 2.011 | 0.5689 | < 0.005*** | 0.1354 |
| **Tyrosine** | 1 | 3.047 | 0.2503 | < 0.005*** | 0.0134* |
| **Glutamate** | 1 | 1 | 0.2411 | < 0.005*** | 0.2656 |
| **Asparatate** | 1 | 2.577 | 0.1373 | 0.0567 | 0.3426 |
| **Asparagine** | 1 | 2.42 | 0.9393 | < 0.005*** | 0.0230* |
| **t4-OH-Proline** | 5.565 | 3.982 | < 0.005*** | < 0.005*** | 0.0625 |
| **Beta-Alanine** | 2.243 | 2.721 | < 0.005*** | < 0.005*** | 0.6251 |
| **Sarcosine** | 1.953 | 2.815 | < 0.005*** | < 0.005*** | 0.5700 |
| **1-methylhistidine** | 1 | 4.327 | < 0.005*** | 0.0715 | < 0.005*** |
| **DHEAS** | 1.209 | 1.534 | < 0.005*** | 0.0384* | 0.9298 |
| **Hexose** | 1 | 1 | 0.0974 | 0.0222* | 0.9327 |
| **3-IPA** | 1 | 1 | 0.0055** | 0.2045 | 0.0980 |
